# Challenges of Generating and Maintaining Protective Vaccine-Induced Immune Responses for Foot-and-Mouth Disease Virus in Pigs

**DOI:** 10.3389/fvets.2016.00102

**Published:** 2016-11-30

**Authors:** Nicholas A. Lyons, Young S. Lyoo, Donald P. King, David J. Paton

**Affiliations:** ^1^The Pirbright Institute, Pirbright, UK; ^2^European Commission for the Control of Foot-and-Mouth Disease, Food and Agriculture Organization of the United Nations, Rome, Italy; ^3^College of Veterinary Medicine, Konkuk University, Seoul, South Korea

**Keywords:** foot-and-mouth disease, pigs, vaccination, immunity

## Abstract

Vaccination can play a central role in the control of outbreaks of foot-and-mouth disease (FMD) by reducing both the impact of clinical disease and the extent of virus transmission between susceptible animals. Recent incursions of exotic FMD virus lineages into several East Asian countries have highlighted the difficulties of generating and maintaining an adequate immune response in vaccinated pigs. Factors that impact vaccine performance include (i) the potency, antigenic payload, and formulation of a vaccine; (ii) the antigenic match between the vaccine and the heterologous circulating field strain; and (iii) the regime (timing, frequency, and herd-level coverage) used to administer the vaccine. This review collates data from studies that have evaluated the performance of foot-and-mouth disease virus vaccines at the individual and population level in pigs and identifies research priorities that could provide new insights to improve vaccination in the future.

## Introduction

Foot-and-mouth disease (FMD) is a viral disease of cloven-hooved animals causing severe economic impacts (1). The disease circulates widely in sub-Saharan Africa and Asia, but has been largely eradicated from South America as well as much of the developed world. It is caused by a Picornavirus (FMD virus: FMDV) that exists as seven immunologically distinct serotypes. Global FMD control efforts are focused at reducing the burden of disease, with the longer-term goal to sequentially eliminate the virus from livestock populations. Vaccination can be a highly effective tool to control FMD, especially when it is implemented together with effective zoo-sanitary measures (farm biosecurity and quarantine) and culling of infected animals. During the 1980s, vaccines were used to effectively eradicate FMD from continental Europe (2), and, more recently, FMD control in South America has employed extensive use of vaccination (3).

In attempts to maximize the impact of limited vaccine resources, most FMD control programs emphasize the use of FMDV vaccines in cattle. As a consequence, many of the published studies that evaluate FMDV vaccine performance have also focused exclusively on their use in cattle. However, some countries have large pig populations that are a major target for FMDV vaccination. The impact of FMD in pigs has recently become particularly important in many Asian countries, such as China and the Republic of Korea, where there have been extensive and sustained FMD outbreaks due to serotype O and A lineages that have emerged from mainland Southeast Asia (4, 5). The continued occurrence of FMD cases in countries that have large pig populations despite extensive vaccination has raised questions about the effectiveness of vaccination in pigs, but published field studies that analyze this issue appear to be lacking. This review highlights the difficulties of FMDV vaccination in pigs at the individual and population level and summarizes the studies that have evaluated the performance of FMDV vaccines in this important domesticated livestock species.

## General Considerations for FMD Vaccination

### Types of Vaccines in Commercial Use Today in Pigs

Foot-and-mouth disease vaccines have been produced on a large scale since the 1940s (6) and are currently manufactured by at least 56 commercial and governmental institutions around the world (Mezzer and Vallée, personal communication, 2014). In all FMDV susceptible species, there are many types of vaccine available, not just by virtue of the serotypes and strains included, but also the adjuvant (aluminum hydroxide/saponin or oil adjuvants as: oil in water, water in oil, and double water emulsion) and inactivation method (binary ethyleneimine or rarely formaldehyde) (7). For pigs, currently available vaccines are formulated with an oil adjuvant, due to poor immunogenicity with the aqueous equivalents, and contain either killed/inactivated FMD virus or a synthetic viral peptide (8, 9).

### Reasons for Vaccine Failure

**Figure 1:**
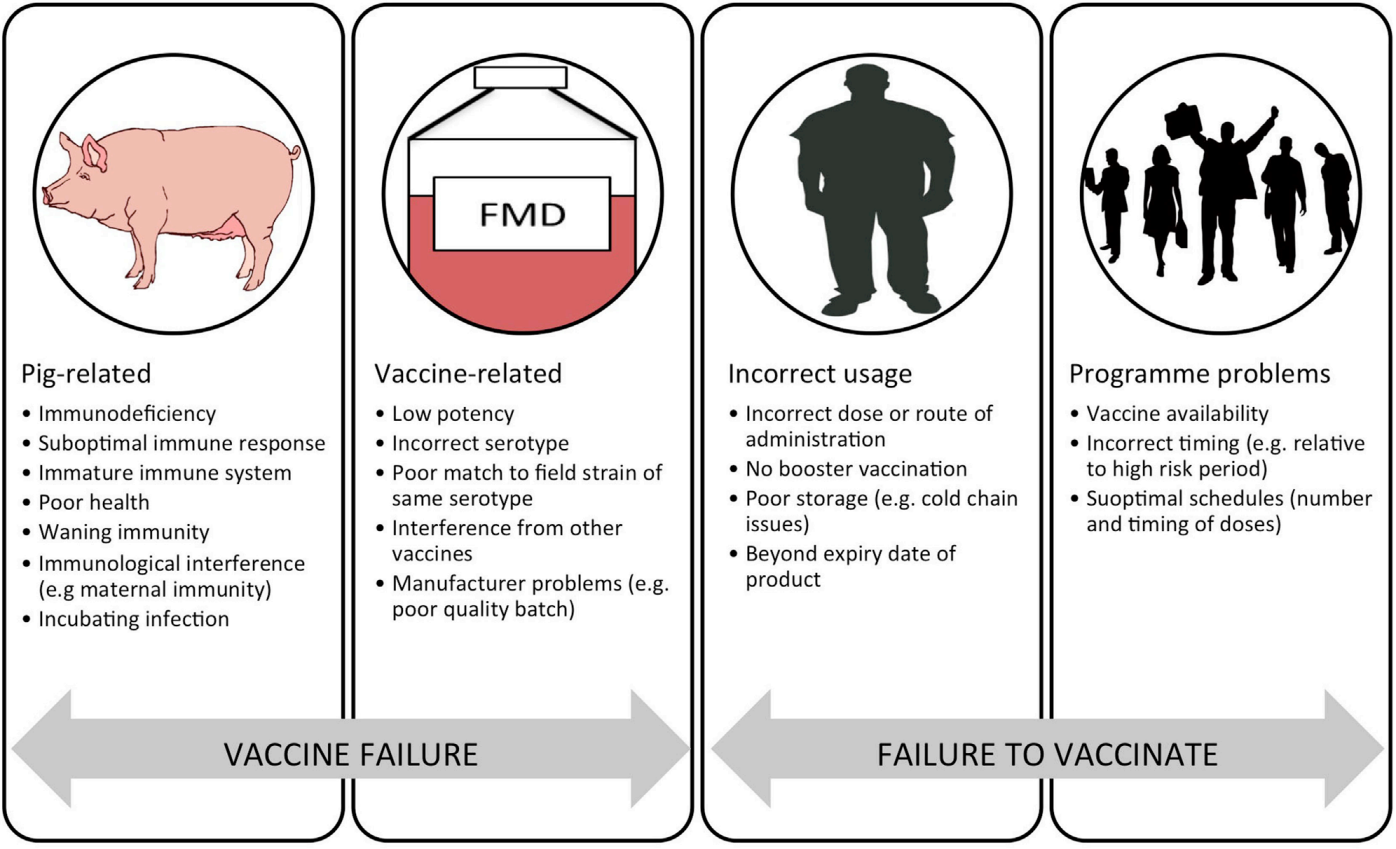
**Schematic representation of the reasons for a failure in vaccination divided into “vaccine failure” and “failure to vaccinate.”** Adapted from Ref. (13).

There are a number of problems with current FMD vaccines that limit their effective use. These include: imperfect antigenic match between the field virus and vaccine strain; variable antigenic payload; antigen instability (principally the 146S virus particles); requirement for a cold-chain; poor adaptation of certain strains for vaccine production; short duration of protection and requirements for repeat boosting; non-sterile immunity with clinically protected animals sometimes becoming infected; high levels of coverage required for herd immunity; and interference by maternally derived antibody (10, 11). Despite these problems, FMD vaccines can play a vital role in disease control and are very widely used, with over two billion doses estimated to be used globally each year (12). The general reasons for vaccination failure have been helpfully summarized by Heininger et al. (13). “Vaccine failure” may be related to the recipient (pig) or the actual vaccine. “Failure to vaccinate” can be due to errors in vaccine use by the user and program-related problems. In the context of porcine FMD vaccines, these key issues are summarized in Figure 1.

## Immunity and Immunogenicity

Comparative interpretation of reports on the evaluation of FMD vaccines are often complicated by significant differences in the potency and other characteristics (e.g., different adjuvants and oil emulsions) of the different vaccines under study, as well as different methods and severity of challenge models (mainly direct or indirect contact with infected unvaccinated or vaccinated donors or intramuscular or intradermal inoculation). The immune responses of pigs to FMD vaccines are less well studied than those of cattle (e.g., details of antibody isotypes, of local immunity, of breadth of antigenic protection, and of the correlation between antibody responses and protection), and there are few field study reports on vaccinated pigs (14). As for cattle and other species, establishing reliable correlates of serological protection for easy interpretation of field studies on vaccine-induced immunity in pigs are hampered by their dependence on specific attributes of the tests, vaccines, and challenge viruses involved.

### Immune Response to FMD Vaccines in Pigs

Inactivated oil-adjuvanted FMD vaccines elicit antibody responses in pigs, and the extent of seroconversion measured by virus neutralization and liquid phase blocking ELISA (LPBE) tests can help to predict clinical protection (15–17). Eblé et al. (18) showed that reduced virus shedding was also correlated to neutralizing antibody levels induced by vaccination and that vaccine-induced mucosal IgA was associated with reduced susceptibility to infection. Cox et al. (19) showed that pigs immunized with high-potency vaccines could be protected against challenge 7 months later, associated with sustained levels of neutralizing antibody and a sustained increase in some cytokine levels in serum (IL-6, IL-8, and in some pigs IL-12). Compared to unvaccinated pigs, vaccinated animals that became infected had lower and shorter lived antibody responses to FMDV non-structural proteins (18, 20).

High potency vaccines can protect pigs by ~4 days after vaccination, before the development of appreciable antibodies (21) and, as for cattle, there appears to be a gray zone where the protection afforded by low levels of antibody is unpredictable (17). This suggests that other factors are involved in protective immunity. Systemic levels of some cytokines have been shown to increase following FMD vaccination in pigs (22, 23), and Rigden et al. (24) showed enhanced chemotaxis of cells of the innate immune defenses. Furthermore, the induction of both cellular and humoral arms of the immune system postvaccination has been demonstrated by measuring Th1 [interferon (IFN) gamma] and Th2 (IL-10) responses (25). Zhang et al. (17) studied cell-mediated immunity in 30 vaccinated and 3 unvaccinated pigs given three different doses of vaccine and challenged intramuscularly with 1000 pig ID50 at 28 days post vaccination (dpv). Twenty-five pigs had antibody levels measured by LPBE that could be associated with protection or not (the gray zone). Protection was associated with vaccine-induced increases in cytotoxic T cell numbers and in levels of IFN gamma, IL-12, and IL-15 in serum. Garcia-Briones et al. (26) reported that a recombinant vaccinia virus expressing the FMDV 3D protein could partially protect pigs through a cell-mediated mechanism in the absence of a humoral antibody response to FMDV.

## Vaccine Potency and Protection

Potency is defined by the OIE as the “concentration of the immunologically active component” (27). Potency according to this definition is often measured by vaccine manufacturers through the quantification of antigen so that a dose of a vaccine delivers a known antigen “payload.” The conventional method of evaluating the effectiveness of FMD vaccines is by experimentally challenging vaccinated and unvaccinated control animals. Although inconsistent with the OIE definition of potency, these evaluations are commonly known as “potency tests.” The first of these tests estimates the 50% protective dose (PD_50_) value and is also the recommended European Pharmacopeia (EP) test. The PD_50_ value is defined as the dose that protects 50% of those under the particular challenge regimen (28). The second OIE-approved test is the “Protection against Podal Generalisation” (PPG) method, which is commonly used in South America.

In the 2009 OIE guidelines, there are descriptions of protocols for calculation of the PD_50_ and PPG based on challenge experiments in pigs which are very similar to those described in cattle. For the PD_50_, three groups of five pigs, no younger than 2 months of age and free of FMD serum antibody, are given either a full dose, quarter dose, or 1/16th dose. They are challenged 28 days later by intradermal inoculation of 10,000 TCID_50_ of the vaccine strain into one of the heel bulbs of the foot. Two unvaccinated control pigs are included for comparison and to demonstrate a consistent phenotype of the challenge strain. For the PD_50_ test, the main difference with the pig protocol is the route of inoculation, as cattle are challenged *via* the intradermolingual route. A PPG equivalent, whereby 16 animals are challenged after receiving a full dose is also described. These descriptions were not included in the 2015 version of this document that states “In general, a successful test in cattle is considered to be sufficient evidence of the quality of a vaccine to endorse its use in other species. Under circumstances where a vaccine is produced for use primarily in a species other than cattle, it may be more appropriate to potency test the vaccine in that same species” (27). Li et al. (29) have proposed an easier approach to inoculation by challenging intramuscularly behind the ear although a suckling mice passaged strain was needed over a conventional cell passaged version. In China, intramuscular inoculation of 1000 pig ID50 of challenge virus is widely used, as described in studies to evaluate novel vaccines (see section below).

### Transmission Studies

There are numerous examples of challenge studies in pigs in the scientific literature to either evaluate the clinical protection afforded by vaccines or their potential role in reducing transmission. Salt et al. (21) evaluated a high potency, oil-based, monovalent serotype C vaccine (strain Oberbayern) by exposing groups of three non-vaccinated or vaccinated pigs to infected animals at 4, 8, 12, 16, and 21 dpv. The challenge virus was homologous to the vaccine strain. Contact was indirect to simulate airborne transmission and looked at both “water-in-oil-in-water” and “oil-in-water” vaccines. All unvaccinated controls showed generalized disease, but all vaccinated animals were protected from clinical disease. Li et al. (29) reported the findings of a homologous PPG test for a serotype O strain using 16 vaccinated pigs and 3 unvaccinated controls that were challenged intramuscularly behind the ear, 28 dpv. All vaccinated animals were protected from clinical disease, and the authors stated that two of the three controls had to show clinical disease for the test to be valid.

Eblé et al. (30) used challenge studies to estimate the impact of vaccination on transmission within pens in a high containment unit using a serotype O Taiwan strain. The vaccine was a double oil emulsion containing 3 μg of 146S antigen per dose. A single animal in a group of six was challenged by intradermal inoculation in the heel bulb, 7 or 14 dpv. Transmission to the in-contact pigs was evaluated by observing clinical signs, seroconversion to NSP antibodies, and detecting virus in oral swabs and serum. Three of the five contact animals in the 7-day group showed generalized clinical disease compared to none of those in the 14-day group. Additionally, no virus could be detected in the 14-day group providing evidence that vaccination can reduce transmission at 14 dpv in this setting. In contrast, a study performed by Parida et al. (20), evaluated transmission and protection at 10 and 29 dpv. The oil-adjuvanted vaccine used was of high potency (>18PD_50_ based on cattle experiments) and contained the O Manisa strain. Challenge was through exposure by direct contact with pigs with clinical disease caused by the O UKG 34/2001 strain of serotype O. Of animals challenged at 10 dpv, 13/16 (81%) were clinically diseased, while in the 29-day group, 2/8 (25%) were affected. In both groups, disease was reported to be milder and associated with reduced virus shedding compared to the unvaccinated control animals. Similar studies by Orsel et al. (31) aimed to assess transmission from infected, vaccinated pigs that had received O Manisa vaccine 14 days before challenge with O/NET/2001. They showed that vaccinated pigs could transmit infection to other vaccinated pigs as readily as to non-vaccinated controls. However, further work by the same group demonstrated that vaccination was able to reduce the transmission between pens (32). The differences reported in these studies could be attributed to different exposure methods, strains, or the small numbers of animals used.

Challenge studies were also performed to evaluate protection from an O Manisa vaccine to a strain from the O Mya98 lineage (33). Vaccines were double oil adjuvanted and >6.0PD_50_ (presumably based on bovine challenge studies although this is not stated). Groups of five pigs were vaccinated and intradermally challenged at either 4 or 7 dpv. A non-vaccinated control group of five animals was also challenged for comparison. All control animals showed generalized disease. Four out of five (80%) animals challenged at 7 dpv were protected compared with three (60%) animals challenged at 4 dpv indicating animals may be protected soon after vaccination. Virus shedding was significantly lower in vaccinated animals compared to controls. Each group was in indirect contact (not physical but shared air handling unit) with five unvaccinated pigs to assess transmission in a controlled environment. No clinical signs or seroconversion was seen in pigs that were in contact with the vaccinated groups despite live virus being detected in the blood. This is in contrast to pigs that were in contact with the unvaccinated control animals although a breach in biosecurity may have explained this contrast. A similar study was performed by the same group using a serotype A Malaysia 97 vaccine and a serotype A/ASIA/Sea-97 lineage challenge strain (relationship value, *r*_1_, around 0.5). Protection from generalized clinical disease was seen in all animals vaccinated 4 and 7 days pre challenge. No disease, FMD antibodies, or live virus was seen in the contact groups, although some animals in contact with the 4-day group were PCR positive on nasal swab (34).

In response to an FMD epidemic in Southeast Asia where there was only a moderate match between field and O Manisa vaccine strains (*r*_1_ around 0.3), Park et al. (35) performed homologous and heterologous challenge studies to evaluate a new vaccine seed strain (O/Andon/SKR/2010). Groups of five, FMD antibody-free, 3-month-old pigs received one of three different antigen payloads (7.5, 10, and 15 μg) in an oil-adjuvanted vaccine and were intradermally challenged 30 dpv with the homologous O/Andon/SKR/2010 strain. Two placebo injected pigs were challenged for comparison. All vaccinated animals were protected from clinical disease, ignoring any lesions seen at the inoculation site. Both control animals had generalized disease. The 10 μg group was subsequently challenged with a heterologous strain of the ME-SA topotype (*r*_1_ value around 0.5), and all animals were protected from clinical disease.

Challenge studies like those described can provide useful information on the potential role of vaccines in FMD control. There is evidence that protection may occur as early as 4 days, and vaccination may reduce transmission. Great care must be taken when extrapolating such results to a population level due to several factors including: variability in effective contact rates and virus shedding (quantity and duration) in the field; exposure routes and doses that have unclear relevance to field conditions; small sample sizes leading to uncertainty in the results from random error; and likely reduced responses to vaccination under program conditions. Therefore, these studies should be complimented by field-based epidemiological studies. Nowadays, decision making on how and when to use vaccination is greatly influenced by simulation studies with computer models. Unfortunately, it is not yet clear how to parameterize such models to make use of the results of potency tests.

## Vaccination Programmes

The level of immunity required to control disease at a population level depends in large part on the basic reproduction number (R_0_) defined as the average number of secondary cases for each primary case in a completely susceptible population. The “effective” reproduction number is the same calculation but in a population with a proportion of immune individuals. If the effective reproduction number is less than one, on average, the circulation of infection will tend to reduce and ultimately cease. On this basis, the herd immunity required to bring the reproduction number to this level (called the “herd immunity threshold” or HIT) can be calculated by

$$\text{HIT}=1-\frac{1}{R_{0}}$$

The basic reproduction number depends on the effective contact rate (i.e., contact between individuals sufficient for transmission per unit time, also known as the transmission parameter), duration of infectiousness, and population size (36). It is possible to estimate the duration of infectiousness from transmission studies although there is likely variation between viral strains and hosts (37). The effective contact rate is likely to be variable depending on environmental factors such as population or stocking density, production systems, season, and nature of any biosecurity practices. There is also the added complexity of population structures and the consideration of transmission at both the within and between herd level (38). R_0_ and the HIT can be estimated using mathematical models, although these should be parameterized as much as possible from field data and tailored to a specific country or region. Small-scale transmission studies can be used to parameterize models, but these should be validated from field-derived data to give greater confidence in model predictions.

The HIT is useful in giving a theoretical target for vaccination coverage (39). In pigs, maintaining sufficient population immunity through vaccination for FMD is a major challenge. Virus transmissibility is potentially high due to higher levels of virus excretion in this species (40), the intensive nature of modern pig production, and a rapid population turnover (particularly in fattening pigs typically slaughtered at 6–7 months old). Additionally, maternal antibodies interfere with the response to vaccines, and there is need for repeated doses of vaccine (discussed in detail in the following section). In some sub-populations with a high transmission risk, a relatively higher vaccination coverage is likely to be required making the case for risk-based vaccination targeting areas of high transmission identified using repeatable epidemiological methods.

### Vaccination Regimes

Table 1 gives two proposed schedules for FMD vaccination in pigs both of which acknowledge the potential impact of maternally derived antibodies (MDA). Experiments have tried to address the issue of MDA interference with vaccination. Francis and Black (41) found that pigs as young as 1 week of age could mount a neutralizing antibody response to vaccine in the absence of MDA. They compared these responses to piglets with MDA from vaccinated sows and found that piglets aged between 1–4 weeks did not show any response with antibodies continuing to decline. An increase was seen in piglets vaccinated at 8 weeks old but was lower in the presence of higher levels of MDA. A recent study by Dekker et al. (42) assessed the serological response to vaccination in piglets at different ages (3–9 weeks) in the presence of MDA. Based on receiving a single dose and neutralizing titers 6 weeks post vaccination, the authors found that vaccination at 7–9 weeks old was optimal. Increases in titers were seen in all age categories although the responses were heavily dependent on the MDA level, which in turn was heavily influenced by the titer in the sow.

**Table 1:** **Recommended schedules for commercially available oil-adjuvanted FMD vaccines licensed for use in pigs**.

Product/Company	Schedules	Source
AFTOPOR (Merial Animal Health)	Once at 2.5 mo (if sporadic FMD cases in area)	“Guidance for Foot and Mouth Disease Vaccination,” Merial Animal Health Limited
	Twice at 2 and 3 mo (Epizootics or highly virulent strain)	
	>2 wo if unvaccinated herd	
DECIVAC (MSD Animal Health)	Young animals with no maternal antibodies: primary dose >2 wo, second dose 6 weeks later in endemic areas. Revaccination 4–6 months later	http://www.msd-animal-health.ph/products/131_118551/ProductDetails_131_118625.aspx
	Young animals with maternal antibodies: primary dose 4–8 wo onward, second dose 6 weeks later in endemic areas, with revaccination 4–6 months later	
	Adults: every 6 months	

*Based on manufacturers listed at http://www.cfsph.iastate.edu/Vaccines/ (accessed August 9, 2016), where the company website states the schedule in English language and specifically for pigs. Both vaccines are licensed for intramuscular injection in the neck region*.

*wo, weeks old; mo, months old*.

Two published studies from Taiwan have attempted to establish the optimal times and schedules for vaccination in pigs using field-derived serological evidence. Chung et al. (43) performed serological surveys as part of an active surveillance strategy on commercial pig farms with a herd size ≥5000. Farms were using an oil-based, >6.0PD_50_ serotype O vaccine. Two dose primary course schedules of 8 and 12 weeks, 10 and 14 weeks, and 12 and 16 weeks were compared through homologous neutralization tests on sera from 97 farms. This suggested that animals vaccinated at 12 and 16 weeks of age had the highest titers and there were significant differences between the vaccine products. This analysis was univariable and did not account for possible confounders and the time between vaccination and sampling. Liao et al. (44) performed a study whereby groups of between 6 and 15 piglets were vaccinated between 2 and 16 weeks old (some groups receiving a booster 4 weeks later). Based on neutralizing titers and homologous challenge studies, both performed at 24 weeks old, the authors suggested the optimum time for the first dose to be 8 weeks of age and titers were not significantly different if the piglet received a second dose at 12 weeks of age. This latter evidence for not needing a second dose based on antibody titers is contrary to the suggested schedules in Table 1.

### Routes of Administration

Both of the vaccines listed in Table 1 are licensed for intramuscular administration in the neck region. Granulomas have been reported to occur in pigs at injection sites post vaccination with water-in-oil adjuvants (45). Although according to McKercher and Gailiunas (45) these were barely visible 6–12 months after vaccination, this could still be a problem in fattening pigs slaughtered at 6–7 months of age where the neck region can have significant value. Such lesions have been reported to occur in 15–20% of pigs but could be easily removed by dissection at slaughter (46). Basarab et al. (47) reported 5/32 (16%) pig carcases had large residual lesions after using a water-in-oil emulsion FMD vaccine requiring extensive dissection. These animals were vaccinated as weaners and the lesions were present at the end of the fattening period although the exact length of time between vaccination and slaughter is not reported. This same study found that intraperitoneal vaccination was equally efficacious to pigs vaccinated intramuscularly based on challenge studies but without the local tissue reaction. An experimental study by Eblé et al. (48) demonstrated that intradermal vaccination at 1/10th the dose of a normal killed vaccine was equally as effective based on challenge studies and neutralizing titers. The small numbers of animals in both of these studies may mean they were statistically underpowered, although both intraperitoneal and intradermal vaccination may offer significant advantages by reducing tissue lesions in fattening animals.

**Figure 2:**
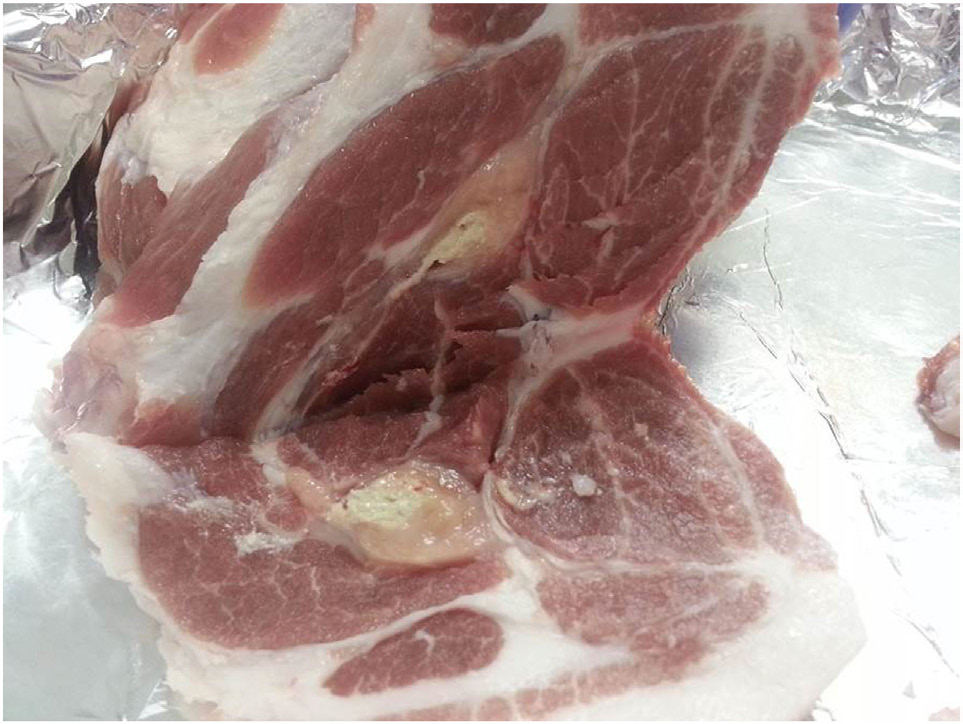
**Gross pathology lesion of an injection-site granuloma in the neck region of a pig from the Republic of Korea**.

The issue of injection-site granulomas post vaccination has been particularly highlighted in the Republic of Korea and has been proposed as an important factor that has contributed to a reduced uptake of vaccination that has compromised coverage. As an example, a recent unpublished survey of 470 fattening crossbred pigs from four commercial farms found visually observable lesions in 87, 80, and 80% at 1, 2, and 3 months post vaccination, respectively. These were visible in live pigs, and all had received a two-dose primary course with the first dose given at 6–8 weeks of age and the second dose 2 weeks later. The injection site was in the neck approximately 2.5 cm caudal to the base of the ear. A subset of animals were slaughtered to demonstrate the gross pathology present as shown in Figure 2.

Although maintaining effective levels of coverage are challenging, a good understanding of the epidemiology will inform targeted vaccination strategies and more effective use of resources. The optimal vaccination schedules will vary depending on the antibody levels in the sow, which in turn will depend on vaccine type and schedules, natural exposure, and other sow- or piglet-related factors. Therefore, it is clear that countries embarking on vaccination programs should perform their own studies to establish optimal vaccination strategies as also suggested by Dekker et al. (42).

## Novel Approaches to Vaccines and Vaccination

Recent years have seen encouraging results with novel FMD vaccines and adjuvants. Those tested in pigs are considered briefly in this review and Table 2 summarizes some of the most promising challenge studies. Peptide vaccines for type O FMDV have been used in China for vaccination of pigs and continue to be improved. More data are needed on the breadth of cross-protection afforded by these vaccines against heterologous virus strains of the same serotype as used for peptide design. New vaccines have been designed, modified, and evaluated based upon FMD virus-like particles (VLP) generated *in vitro* or in the vaccinated pig through expression by virus vectors, especially adenoviruses. Specific methods of attenuating live FMDV now show considerable promise for overcoming the problem of combining inocuity with immunogenicity and can provide protection within 2 days. Data on duration of protection are awaited. IFNs and IFN inducers can not only provide extremely rapid and serotype non-specific protection against FMDV but they can also enhance the protection afforded by specific FMDV antigens and reduce the doses of adenovirus-vectored vaccines required for protection. New adjuvants have mostly been tested as additional incipients for oil-based vaccines and properly controlled and powered comparative studies of different adjuvants have not been published. There have been few recent studies of mucosal vaccine targeting or to evaluate DNA vaccines.

**Table 2:** **Selected pig challenge study results with promising outcomes for novel vaccines**.

Vaccine	Vaccination^a^	Challenge	Protection	Reference
Live FMDV A12 attenuated by L^pro^ mutation (A12-SAP)	15 pigs vaccinated with 10^5^, 10^6^, or 10^7^ pfu A12-SAP by subcutaneous injection	Intradermal heel bulb inoculation with 10^5^ FMDV A12 at 21 dpv	All 15 pigs protected against clinical signs (fever or vesicles), viremia, and nasal shedding	(49)
	9 pigs vaccinated with 10^6^ pfu A12-SAP by subcutaneous injection	Intradermal heel bulb inoculation with 5 × 10^5^ FMDV A12 at 2, 7, or 14 dpv	8 of 9 pigs protected against clinical signs	
Adenovirus vector expressing FMDV A24 P1-2A, 2B, 3B, 3C with Poly ICLC adjuvant in PBS	6 pigs vaccinated with 2.5 × 10^6^ vector plus 1 mg poly ICLC by subcutaneous injection of 2 ml dose at 2 sites (other vaccination schedules evaluated)	Intradermal heel bulb inoculation with 10^5^ FMDV A24 at 7 or 21 dpv	All 3 pigs challenged at 21 dpv protected against clinical signs, viremia, and nasal shedding (partial protection when challenged at 7 dpv)	(50)
Adenovirus vectors, one expressing porcine alpha and gamma interferons and the other expressing 3 small interfering RNAs	15 minipigs vaccinated with 7.2 × 10^9^ or 1.75 10^10^ TCID_50_ of a combination of the adenovirus vectors (1:5 ratio of Ad-IFN titer to Ad-3siRNA titer) by intramuscular injection (other vaccination schedules evaluated)	Direct contact of 5 groups of 3 “vaccinated” minipigs at 2, 4, and 7 dpv, for 18 h with donor minipigs infected with FMDV strain O/Andong/SKR/2010	At the low “vaccine” dose, complete clinical protection in 2/3, 1/3, and 0/3 minipigs at 2, 4, and 7 dpv. At the high “vaccine” dose it was 3/3 and 1/3 at 4 and 7 dpv. Viremia and oral shedding also reduced or prevented in some minipigs	(51)
FMDV multi-epitope (B and T cell) from 4 FMDV O topotype viruses with poly IC adjuvant. VP1 epitopes from O/Mya/98, O/HN/CHA/09, O/Tibet/99, O/IRN/2010. Two universal (non-FMDV) T cell epitopes	45 pigs vaccinated in three groups of 15 pigs, each group consisting of 3 subgroups of 5 pigs receiving different doses: full (2 ml), 1/3, or 1/9 dose by volume intramuscularly. The full dose contained 300 μg of epitope protein and 300 μg poly IC	Three potency tests involving challenge at 28 dpv by intramuscular inoculation with 1000 50% infectious doses of one of three FMDV O strains: O/Mya/98, O/HN/CHA/93, O/Tibet/99	PD_50_ results were 15.6 (O/Mya/98 challenge), 15.6 (O/HN/CHA/93), and 7.0 (O/Tibet/99)	(52)
Pseudorabies virus expressing P1-2A, 3C from FMDV O/ES/2001 (PRV-P12A3C)	5 pigs vaccinated with 10^6^ TCID_50_ PRV-P12A3C in 2 ml by intramuscular injection with identical booster at 21 dpv	1000 50% infectious doses of FMDV O/OR/80 by intramuscular inoculation at 15 days after booster vaccination	3 of 5 vaccinated pigs fully protected against clinical signs	(53)
FMDV Asia1/Jiangsu/China/2005 VLP produced in *E coli* as SUMO-VP0/VP1/VP3 fusion proteins, subsequently purified and cleaved	5 pigs vaccinated with 50 μg VLP in oil adjuvant by intramuscular route	1000 50% infectious doses of FMDV Asia1/Jiangsu/China/2005 by intramuscular inoculation	All 5 vaccinated pigs fully protected against clinical signs	(54)
Dendrimeric B and T cell epitopes from FMDV O/UKG/11/2001	6 pigs vaccinated twice 21 days apart with 2 ml oil adjuvant containing 2 mg peptide by intramuscular route (other related vaccines evaluated)	1.6 × 10^4^ FMDV O/UKG/11/2001 by heel bulb inoculation at 18 days after second vaccination	All 6 vaccinated pigs fully protected against clinical signs and for 5 of 6 pigs no virus shedding detected in pharyngeal or nasal swabs	(55)

*^a^All studies included control mock or unvaccinated pigs, and some studies included comparison with conventional vaccines, but details not given here*.

### Adenovirus-Vectored Vaccines

Adenovirus-vectored FMD vaccines conditionally licensed in the USA in 2012 for use in cattle, have also shown efficacy in pigs. A replication-defective human serotype 5 adenovirus expressing the capsid encoding genes and the 3C protease needed for their cleavage and incorporating genetic material from the A24 strain of FMDV was given to pigs at a dose of 5 × 10^9^ pfu, resulting in complete clinical protection against homologous FMDV by contact challenge at 7, 14, and 42 dpv (56). It was later shown that a modified vector insert also expressing the FMDV 2B gene improved the early antibody response to the FMDV capsid (57).

The same adenovirus vector when administered at high doses can deliver IFNs to provide early protection against FMDV and types I, II, and III IFN given this way can all provide protection to pigs for up to 5 days with evidence of synergistic action between different IFN types [reviewed by Stenfeldt et al. (58)]. Patch et al. (59) explored the possibility of selecting for a cytotoxic T cell response to FMDV in pigs vaccinated with an adenovirus expressing an inefficiently cleaved capsid precursor, but the protective value of this was not reported.

Kim et al. (51) developed recombinant adenoviruses for the simultaneous expression of porcine alpha and gamma IFNs as well as three small interfering RNAs targeting FMDV mRNAs encoding non-structural proteins. The antiviral effects of these vectors were synergistic in porcine cells, suckling mice, and minipigs. The vectors administered at high dose by the intramuscular route fully protected 3 pigs against an 18-h direct contact challenge 1 day later. Partial protection at challenge 2–4 days after administration was mostly lost at 7 days. *In vitro*, the combination treatment was effective against all serotypes of FMDV.

### Other Vectored Vaccines

Canine adenovirus type 2 expressing VP1 elicited low levels of FMDV neutralizing antibody in pigs (60). A recombinant pseudorabies virus expressing the capsid and 3C encoding genes of FMDV serotype O partially protected (3 of 5) pigs against an intramuscular challenge with 1000 ID_50_ of a heterologous live type O FMDV [(53); Table 2]. An earlier pseudorabies virus vector expressing only VP1 of FMDV was less effective (61).

Yang et al. (62) reported the insertion of VP1 T and B cell epitopes of FMDV serotype O into a bamboo mosaic virus (BMV), resulting in expression of a fusion protein. Pigs inoculated intramuscularly with 5–10 mg of the recombinant BMV in a mineral oil adjuvant produced VP1-specific cell-mediated immunity and neutralizing antibodies. The protection of pigs against challenge with live FMDV was described after a double dose of the recombinant BMV, and protection was said to be possible after one dose.

Recombinant baculoviruses were used by Crisci et al. (63) to generate chimeric virus-like particles of rabbit haemmorhagic disease virus fused to a FMDV T cell epitope from the 3A viral non-structural protein. Intramuscular inoculation of pigs with this chimera and an oil adjuvant generated FMDV-specific cell-mediated immunity and antibodies.

### Interferons

Polyriboinosinic-polyribocytidylic acid stabilized with poly-l-lysine and carboxymethyl cellulose (poly ICLC) is a synthetic double-stranded RNA (dsRNA) that is a viral mimic and activates multiple innate immune pathways through interaction with toll-like receptor 3 and MDA-5. It is a potent inducer of IFNs and can protect against FMD at 1 day after treatment (64). Its adjuvant affect on FMD vaccines in pigs was reported 40 years ago (65). Recently, it was shown to reduce, by 80-fold, the dose required for protection of a recombinant adenovirus expressing FMDV A24 capsids [(50); Table 2]. Another synthetic analog of dsRNA, polyinosinic-polycytidylic acid (poly IC), potentiated the protection afforded by a multi-epitope vaccine in pigs (66). This vaccine incorporated linked B cell epitopes (the G–H loop and C terminus of VP1) from four topotypes of serotype O flanked by two universal T cell epitopes. The final product in an oil adjuvant with poly IC protected pigs with 50% protection values of 7–16 against different challenge viruses [(52); Table 2].

### Other Adjuvants

Barrette et al. (67) showed that intranasal immunization of pigs with detoxified *Escherichia coli* enterotoxins LTK63 and LTR72 linked to a peptide derived from the FMDV serotype O1-BFS VP1 G-H loop enhanced the antigen-specific mucosal and systemic immune responses to FMDV. Guo et al. (68) reported that a CpG-enriched plasmid enhanced the efficacy of a conventional FMD killed vaccine. Park et al. (35, 69) vaccinated groups of five pigs with a conventional FMD vaccine antigen plus either the oil adjuvant used in the Republic of Korea or with novel adjuvants (Carbigen, Emulsigen-D and ISA 201). In terms of immune response and post-challenge protection, the novel antigens were at least as good.

In a small field trial, administering 60 mg of poly gamma glutamic acid (PGA) 3 days before FMDV vaccination of young pigs resulted in slightly more animals with detectable levels of FMDV antibodies 2–6 weeks later (70). Li et al. (71) reported increased antibody responses of pigs to a conventional FMDV vaccine supplemented with ginseng stem and leaf saponins. Xiao et al. (72) showed that an extract of the seeds of *Momordica cochinchinensis* (Lour.) Spreng. (ECMS) had a synergistic effect in improving the immune response of pigs after vaccination with inactivated FMDV antigens in an oil emulsion vaccine.

### Live Attenuated Vaccines

Deleting the Lpro gene of FMDV A12 gave rise to an attenuated virus that partially protected pigs against wild-type challenge (73). Meanwhile, FMDV A24 lacking Lpro but with a capsid substituted from serotype O was still somewhat virulent for pigs. Changing the capsid genes to those of a cell culture adapted virus eliminated the virulence, but the resulting virus did not protect pigs when used as a vaccine (74). In contrast, mutating a conserved protein domain within the Lpro gene of FMDV A12 gave rise to a virus that was avirulent in pigs at a dose of 10^7^ but nevertheless elicited protection against FMDV challenge from 2 dpv (49).

Codon bias deoptimization of the FMDV capsid-coding region (P1) introduced 489 nucleotide changes (19%) but retained virus viability. The vaccine safety margin was ~1000-fold higher for pigs than for wild-type virus. Consistently, high levels of antibody titers were induced, even at the lowest dose tested (75).

### Protein/Peptide Vaccines

Shao et al. (76) reported on the further development of a tandem repeat multiple-epitope recombinant vaccine against FMDV serotype O containing three copies of two VP1 epitopes of the O/China/99 strain of FMDV coupled with a porcine IgG heavy-chain constant region (77). This peptide vaccine elicited high titers of FMDV specific antibodies in pigs at 30 dpv and conferred complete protection against a challenge with 1000 50% infective doses of the O/China/99 strain. Trials of another B cell epitope vaccine (52) have already been described above under IFNs (Table 2). Dong et al. (78) inserted the coding sequences of a FMDV serotype O VP1 epitope into a coliphage, resulting in an epitope-phage recombinant protein that formed a virus-like particle (VLP). Challenge inoculation of twice vaccinated pigs with the live homologous virus resulted in three of five animals being clinically protected from FMD.

Building upon earlier work (79, 80), Blanco et al. (55) reported that a synthetic dendrimeric peptide vaccine comprising two copies of a FMDV VP1 B cell epitope linked to a FMDV 3A T cell epitope protected pigs against disease and virus shedding after two doses of vaccination followed by challenge inoculation with live homologous FMDV O UK 2001 (Table 2). Guo et al. (54) have developed a bacterial expression system to generate VLPs of the FMDV Asia 1 capsid proteins. The FMDV genes VP0, VP1, and VP3 were each expressed as fusion products with the small ubiquitin like modifier protein (SUMO) and after removal of the SUMO moiety, the FMDV proteins assembled into VLPs. Five pigs vaccinated with 50 μg of VLP emulsified in oil adjuvant were fully protected from challenge inoculation with live homologous FMDV (Table 2).

### DNA Vaccines

DNA vaccines have not been completely effective in livestock despite promising results in mice. Multiple doses of plasmids expressing FMDV proteins or epitopes with coexpression of immunostimulants, and/or with conventional antigen boosters have been required to protect pigs against FMD (81–83). Most recently, Borrego et al. (84) reported partial protection of pigs after three immunizations with a DNA vaccine encoding FMDV B and T cell epitopes fused to the variable fragment of a mouse immunoglobulin against Class II swine leukocyte antigens.

### Mucosal Vaccines

Although mucosal IgA may be elicited by parenteral immunization routes [e.g., Ref. (80)], mucosal vaccination might help to block FMDV entry. Barrette et al. (67) evaluated detoxified *Escherichia coli* enterotoxins LTK63 and LTR72 as mucosal adjuvants showing enhanced antigen-specific mucosal and systemic immunity for non-replicating antigens, including FMDV, upon intranasal immunization in pigs. Song et al. (85) reported vaccination of pigs with a recombinant VP1 epitope complex of serotype O FMDV fused to the cholera toxin B subunit (hCTB). Eight of ten pigs that were given three intraperitoneal immunizations were protected from challenge by inoculation with 106.5 TCID_50_ type O FMDV. Wang et al. (86) showed that intranasal delivery of cationic PLGA nano/microparticles loaded with various FMDV DNA vaccine formulations encoding IL-6 as a molecular adjuvant enhanced protective immunity against FMDV, particularly pc-IL2AP12A3C with the IL-6 gene located before the P12A3C gene. Nevertheless, only partial protection against challenge with FMDV was achieved in pigs.

### Chimeric Killed Vaccines

Blignaut et al. (87) produced a killed vaccine from a chimeric virus in which the capsid encoding genes were replaced with those from a different serotype. The resulting SAT 2 FMDV with a SAT 1 capsid were used to make a conventional killed vaccine that was potency tested in 17 pigs (three groups of five pigs given different vaccine doses and two unvaccinated control pigs). After a SAT 1 challenge by heel bulb inoculation, the PD_50_ was found to be >6.4. Zheng et al. (88) substituted the capsid-encoding region of a serotype A virus vaccine for a more recent field isolate to update the antigenic match. The new vaccine was shown to protect against both the homologous strain and another semi-heterologous one.

## Research Priorities

This review summarizes studies that have been undertaken to evaluate the performance of FMD vaccines in pigs, as well as introduce novel vaccination strategies that might be employed for FMD control in the future. Collectively, these data provide a valuable body of evidence that are especially relevant in the parts of the world where pigs play a central role in the maintenance and spread of the virus. Although a number of these experimental studies have evaluated the performance of FMDV vaccines, it is apparent that field data for such evaluation in pigs are currently lacking. Furthermore, much of this work is dependent upon bovine reagents, such as antigenic profiling (vaccine-matching), or exploits *in vitro* measurements of “correlates of protection” derived from cattle studies. In view of this paucity of data, when using vaccines in these settings, it is important to consider the different factors that influence whether, or not, a vaccine is likely to be efficacious. These include the (i) regime used (timing and frequency of vaccination); (ii) potency and formulation of oil vaccines; and (iii) antigenic match between the vaccine and circulating field strain. Although these three points are often assessed (and discussed) separately, they have an intimate relationship that underpins the performance of a vaccine. For example, it is usually accepted that a less than perfect antigenic match can be compensated by administration of a high potency vaccine; however, the impact of vaccine regime (as well as the herd-level coverage) is often ignored. In order to improve vaccine-induced immune responses, additional areas that warrant further scientific investigation include more systematic research to evaluate alternative vaccine adjuvants for vaccination in pigs, and research to validate of alternative routes (IM, IP, SC, ID) and sites of vaccination (to minimize local tissue granulomas in valuable meat cuts) and even multiple sites (with a divided dose). Effective (improved) vaccination regimes are also necessary to generate optimum protection in pigs to accommodate maternal antibody responses (to reduce the immunity gap).

Data from recent field outbreaks in Asia highlight the challenges posed by the control of FMD in pigs. While initiatives to improve the quality of vaccines and coverage that are tailored for pigs have the potential to make a positive impact on FMD control, it should be remembered that vaccination-alone is not a magic panacea and that FMD control, especially in the face of high amounts of circulating virus, is often reliant upon the implementation of effective zoo-sanitary (bio-containment) measures, as well as the maintenance of adequate local veterinary resources so that new clinical cases are rapidly investigated and detected.

## Author Contributions

NL, DK, and DP wrote extensive sections of the manuscript. NL led the structuring and format of the review. YL provided unique insights and data from the FMD situation in the Republic of Korea (the section of granulomas with image). All the authors read, edited, and approved the final manuscript.

## Conflict of Interest Statement

The authors declare that the research was conducted in the absence of any commercial or financial relationships that could be construed as a potential conflict of interest.
